# Dietary starch levels modulate production performance and whole-body nutrient metabolism in dairy cows

**DOI:** 10.3168/jdsc.2025-0902

**Published:** 2026-01-16

**Authors:** Usman Arshad, Martin Huser, Mario A. Barrientos-Blanco, Md Zakirul Islam, Xiaoqi Ma, Rong Peng, Kai Wang, Mutian Niu

**Affiliations:** Animal Nutrition, Institute of Agricultural Sciences, Department of Environmental Systems Science, ETH Zürich, Zürich 8092, Switzerland

## Abstract

The objective of this study was to evaluate the effects of increasing dietary starch concentrations on production performance and whole-body nutrient metabolism in lactating dairy cows. Twelve multiparous Holstein cows at mean (±SD) 89 ± 13 DIM were assigned to a replicated 3 × 3 Latin square design comprising three 21-d periods. Each period consisted of 14-d dietary adaptation followed by 7 d of data and sample collection. Treatments consisted of low-starch (LS; 9.66% of DM), medium-starch (MS; 20.6% of DM), and high-starch (HS; 29.1% of DM) diets. Crude protein concentrations were 15.9%, 14.7%, and 14.7%, and NDF concentrations were 47.9%, 38.3%, and 36.6% for the LS, MS, and HS diets, respectively. Dry matter intake and milk yield and composition were measured throughout the sampling week. Gas exchange including CH_4_, O_2_, and CO_2_ were measured using a head chamber (GreenFeed system) on the last day of the sampling period for the calculation of respiratory quotient, net carbohydrate oxidation (COX_net_), net fat oxidation (FOX_net_), and metabolic heat production (mHP). Least squares means and SEM are presented in the sequence of LS, MS, and HS. Increasing dietary starch concentration linearly increased net energy intake (38.9 vs. 40.3 vs. 40.7 ± 0.87 Mcal/d) and milk yield (33.5 vs. 36.6 vs. 38.9 ± 1.24 kg/d), and tended to linearly increase ECM yield (39.1 vs. 43.5 vs. 43.1 ± 1.50 kg/d), without a concurrent increase in DMI or BW loss, resulting a tendency for improved feed efficiency (ECM/DMI; 1.62 vs. 1.77 vs. 1.78 ± 0.06 kg/kg) in dairy cows. Yield of milk true protein increased linearly (1.17 vs. 1.37 vs. 1.34 ± 0.06 kg/d), whereas MUN concentrations decreased linearly (18.1 vs. 12.9 vs. 10.4 ± 0.56 mg/dL) with increasing dietary starch. Respiratory quotient tended to respond quadratically, remaining similar in LS and MS but higher in HS-fed cows (0.90 vs. 0.90 vs. 0.94 ± 0.01), indicating a shift in substrate oxidation toward greater carbohydrate utilization. Correspondingly, increasing dietary starch concentration linearly increased COX_net_, both in absolute (5,432 vs. 5,848 vs. 7,276 ± 366 g/d) and metabolic BW (mBW) adjusted terms (39.0 vs. 41.6 vs. 52.0 ± 2.6 g/kg mBW). In contrast, FOX_net_ responded quadratically, peaking in MS both in absolute (742 vs. 906 vs. 450 ± 150 g/d) and mBW-adjusted terms (5.28 vs. 6.42 vs. 3.29 ± 1.07 g/kg mBW). Consistently, mHP increased linearly with starch concentration, both in absolute (34.8 vs. 37.4 vs. 38.1 ± 0.73 Mcal/d) and mBW-adjusted terms (0.25 vs. 0.26 vs. 0.27 ± 0.01 Mcal/kg mBW), indicating greater energy expenditure. Overall, these findings indicate that increasing dietary starch shifted whole-body substrate oxidation toward carbohydrates and improved energy utilization and feed efficiency in mid-lactation dairy cows.

## Highlights

• Increasing dietary starch improved feed efficiency in lactating cows.
• Increasing starch content shifted substrate oxidation toward carbohydrates over fat.
• Fat oxidation decreases, and metabolic heat production increases at high starch.

## Graphical Abstract

**Figure:**
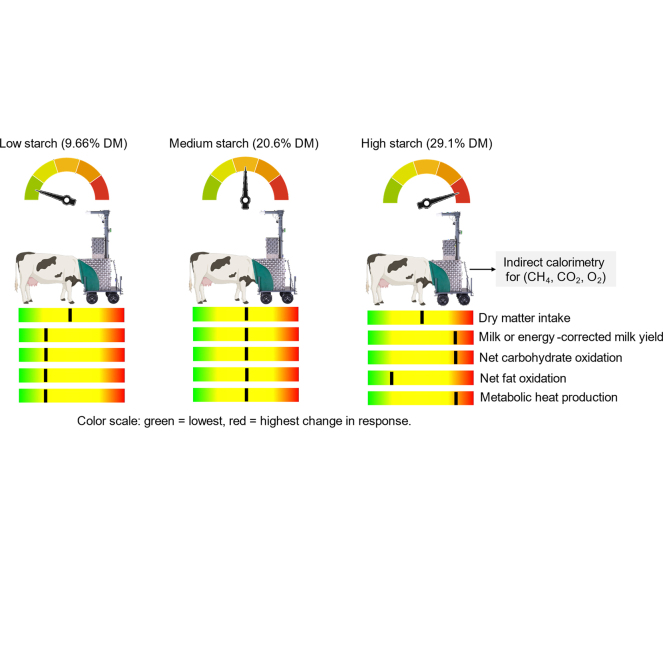
**Summary:** The objective of this study was to evaluate the effects of increasing dietary starch concentrations on production performance and whole-body nutrient metabolism in lactating dairy cows. Twelve multiparous mid-lactation Holstein cows were assigned to a replicated 3 × 3 Latin square design with three 21-day periods, each consisting of 14 days of dietary adaptation followed by 7 days of sampling. Cows were fed low-, medium-, or high-starch diets, and dry matter intake (DMI) and milk production were recorded, whereas gas exchange was measured using a head chamber (GreenFeed system) to estimate net carbohydrate (COX_net_) and fat oxidation (FOX_net_), and metabolic heat production (mHP). Increasing dietary starch enhanced production performance and feed efficiency without affecting DMI, and shifted whole-body substrate oxidation toward carbohydrates, resulting in increased COX_net_, reduced FOX_net_, and higher mHP. Overall, higher dietary starch improved energy utilization by favoring carbohydrate oxidation over fat, supporting greater feed efficiency in lactating dairy cows. DM = dry matter

Optimizing nutrient utilization in lactating dairy cows is essential for improving production efficiency and reducing the environmental footprint of dairy farming. Among dietary macronutrients, starch is a major determinant of ruminal fermentation patterns, systemic energy metabolism, and milk synthesis in ruminants (Huntington, 1997; Boerman et al., 2015). As a rapidly fermentable carbohydrate in the rumen, it is plausible to speculate that dietary starch may influence the balance between net carbohydrate oxidation (**COX_net_**) and net fat oxidation (**FOX_net_**), with potential implications for the partitioning of energy toward milk synthesis and maintenance. Investigating these systemic metabolic effects requires assessment of gas exchange and nutrient oxidation in animals.

Indirect calorimetry via respiration chambers is considered the “gold standard” for assessing whole-animal energy metabolism by quantifying O_2_ consumption, CO_2_ and CH_4_ emissions, and metabolic heat production (**mHP**; Troy et al., 2016). These measurements enable detailed insights into nutrient oxidation and energy partitioning. Technological advances such as GreenFeed (**GF**) system, a head chamber technique, have provided noninvasive, real-time assessments of gas exchange, allowing metabolic measurements under more natural and less restrictive feeding environments (Hristov et al., 2015). Such measurements may support investigations into how dietary interventions influence systemic substrate oxidation, mHP, and nutrient partitioning in lactating cows. The present experiment aimed to evaluate the effects of increasing dietary starch concentrations on whole-animal nutrient metabolism using GF in lactating dairy cows. We hypothesized that increasing dietary starch content would alter mHP and substrate oxidation patterns, ultimately affecting nutrient partitioning relevant to lactational performance and feed efficiency in dairy cows.

All experimental processes involving live animals were approved by the Cantonal Veterinary Office of Kanton Zürich (ZH150/2024). The experiment was conducted with dairy cows at the AgroVet-Strickhof Farm (Lindau, Switzerland). Twelve multiparous lactating cows (mean ± SD: lactation number 3.50 ± 1.17; 89 ± 13 DIM; 36.8 ± 3.5 kg/d milk yield; 726 ± 48 kg BW) were assigned to a replicated 3 × 3 Latin square design with 21-d periods. Cows were blocked into squares (n = 4) based on their 7-d average milk yield measured before the start of the experiment to ensure balanced production levels across treatments. Each period included 14 d of dietary adaptation followed by 7 d of sample collection. Treatments consisted of low-starch (**LS**; 9.66% of DM), medium-starch (**MS**; 20.6% of DM), and high-starch (**HS**; 29.1% of DM) diets. The rationale for using a wide range of dietary starch concentrations was to create a broad physiological gradient that would allow us to capture metabolic adaptations across different levels of starch availability. The ingredients and chemical composition of the diets are presented in Table 1. The total duration of the experiment was 63 d. During the first 14 d of each experimental period, the cows were housed in a freestall barn with wheat straw bedding and equipped with a feeding trough (Waagen Döhrn GmbH, Germany) and a Calan-gate system (American Calan Inc., Northwood, NH) for individual feeding. The diet was fed ad libitum at 110% of the previous day's intake, and the TMR was mixed before each morning feeding. Cows were milked twice daily, at 0530 and 1530 h, and their milk yield was recorded by the system. Cows were fed twice daily at 0700 (60%) and 1600 (40%) h. On d 15, cows were moved to a tiestall barn with individual access to drinking water and individual feeding plates (Mettler Toledo GmbH, Greifensee, Switzerland), and samples were collected. Samples of individual feedstuffs and TMR were collected in each sampling week and processed for DM and ingredients composition. Milk yield and DMI were monitored daily throughout the sample collection periods. A total of 4 milk samples per period were collected for composition analysis: the evening of d 5, the morning and evening of d 6, and the morning of d 7 of the sampling week. Milk samples were preserved and analyzed for composition. Milk urea N was calculated as urea concentration (mg/dL) × 0.466 and milk true protein was calculated as milk CP (%) − MUN (mg/dL) × 6.38/1,000. Yield of ECM was calculated according to NASEM (2021): [(12.82 × fat yield) + (7.13 × true protein yield) + (0.323 × milk yield)], and feed efficiency was calculated as the ratio of ECM yield to DMI. Body weight was recorded at the start and end of each period of the experiment.

**Table 1:** Ingredient and nutrient composition (mean ± SD^1^) of diets fed to mid-lactating Holstein cows

Item	Treatment diet^2^		
	LS	MS	HS
Ingredient,^3^ % of DM			
Grass silage	23.7	37.1	35.1
Corn silage	16.8	4.77	9.50
Alfalfa haylage	36.9	16.4	4.53
UFA-249	7.38	7.96	11.3
UFA-173	7.75	8.95	9.96
Mineralized concentrate	7.38	7.10	3.62
Ground corn, dried	—	17.7	26.0
Diet composition, DM basis			
NEL,^4^ Mcal/kg of DM	1.60	1.62	1.65
DM, %	57.4 ± 1.07	55.1 ± 1.01	54.4 ± 1.04
CP, %	15.9 ± 0.49	14.7 ± 0.64	14.7 ± 0.99
NDF, %	47.9 ± 3.22	38.3 ± 1.26	32.9 ± 0.95
Forage NDF,^4^ %	42.5	33.8	27.2
ADF, %	31.0 ± 1.15	25.8 ± 0.95	21.1 ± 2.10
Starch, %	9.66 ± 0.07	20.6 ± 1.21	29.1 ± 1.71
Fatty acids,^4^ %	1.83	2.20	2.53

1 Means of 3 composite samples analyzed.

2 Mid-lactating Holstein cows were fed diets containing low starch (LS), medium starch (MS), or high starch (HS).

3 UFA-249 and UFA-173 (UFA, Sursee, Switzerland), and mineralized concentrate (no. 50109, Getreidesammelstelle, Thalheim, Switzerland; % DM basis) contained DM = 86.9, 88.0, 90.0; NDF = 9.00, 15.9, 2.60; ADF = 24.4, 7.40, 1.00; CP = 39.0, 27.3, 34.16; starch = 17.0, 32.4; 17.0; crude fat = 5.50, 8.50, 3.19; and ash = 6.50, 7.88, 7.89, respectively.

4 Calculated using the NASEM Dairy (NASEM, 2021) according to the chemical composition of the dietary ingredients and adjusted for observed DMI for each treatment.

Gas exchange data, including CH_4_, CO_2_, and O_2_, were measured with the GF system (C-Lock Technology Inc., Rapid City, SD) in a tiestall barn. Gas samples were collected on the last day of sampling week in each period at 1000, 1600, 2200, and 0400 h. The system was equipped with an automated collection unit to capture gas samples with a default sample flow rate of 1 L/min operated by a rotameter (10A6130 Glass Tube Purge and Low Flow Meters, Dwyer Instruments Inc.). Each gas collection lasted 5 min, with the GF system collecting background barn air for 2 min between cows to flush the system. During each collection, the cow received a maximum of 8 drops of bait feed, each weighing 30 g, in the form of pellets (UFA-118, UFA, Sursee, Switzerland). Gas exchange data were collected only when proximity sensors confirmed that the cow's head was inside the GF head chamber, and gases were monitored in real time using the Control Feed mobile application (C-Lock Technology Inc., Rapid City, SD) as described previously (Barrientos-Blanco et al., 2025). All parameters related to metabolic gas exchange and nutrient oxidation were calculated based on the average values of gas measurements collected across 4 times during the sampling week of each period. The concentrations of CH_4_ and CO_2_ measured using GF were recorded in grams per day and subsequently converted to liters per day using their respective molecular weights and gas constants. Specifically, CO_2_ (g/d) and CH_4_ (g/d) were converted to volume (L/d) using the molecular weight of each gas and the molar volume at standard temperature and pressure (22.4 L/mol), assuming ideal gas behavior. Fermentative CO_2_ (fCO_2_) was calculated according to Chwalibog et al. (1996) as

fCO_2_ (L/d) = 1.70 × CH_4_ (L/d),

whereas metabolic CO_2_ (mCO_2_) was determined by subtracting fCO_2_ from total CO_2_ production as reported (Chwalibog et al., 1996; Kennedy and Kuhla, 2023):

mCO_2_ (L/d) = total CO_2_ (L/d) − fCO_2_ (L/d).

The respiratory quotient (**RQ**) was calculated as the ratio of metabolic CO_2_ to O_2_ consumption over a 24-h period as reported (Kennedy and Kuhla, 2023):

RQ = mCO_2_ (L/d)/O_2_ (L/d),

where the COX_net_ and FOX_net_ were calculated using stoichiometric equations previously described by Kennedy and Kuhla (2023):

COX_net_ (g/d) = 4.57 × mCO_2_ (L/d) − 3.23 × O_2_ (L/d) − 2.60 × urinary N (g/d),

FOX_net_ (g/d) = 1.69 × O_2_ (L/d) − 1.69 × mCO_2_ (L/d) − 2.03 × urinary N (g/d).

Heat production (**HP**) was estimated based on observed O_2_ consumption and total CO_2_ production, corrected for CH_4_ production and urinary N, using Brouwer's (1965) equation:

HP (Mcal/d) = 0.003866 × O_2_ (L/d) + 0.001200 × CO_2_ (L/d) − 0.000518 × CH_4_ (L/d) − 0.001431 × urinary N (g/d),

and urinary N was calculated according to the Huhtanen et al. (2015) using following equation:

urinary N (g/d) = 4.87 × MUN (mg/dL) + 1.59 × milk yield (kg/d) + 0.149 × BW (kg) − 27.

Mean ± SD values of CH_4_ (492 vs. 515 vs. 440 ± 59.4 g/d), CO_2_ (14,403 vs. 15,394 vs. 15,780 ± 1,243 g/d), O_2_ (9,850 vs. 10,601 vs. 10,726 ± 847 g/d), and urinary N (222 vs. 203 vs. 194 ± 15.8 g/d) were observed or used in LS-, MS-, and HS-fed cows, respectively. All metabolic outputs such as COX_net_, FOX_net_, and HP were further normalized to metabolic BW (**mBW**), defined as BW^0.75^, and expressed both in absolute values and per kilogram of mBW.

A priori power analysis was performed using the *pwr4exp* package in R (version 4.5.1, R Core Team) following Wang et al. (2025). Three dietary starch levels (LS, MS, HS) were evaluated using preplanned orthogonal polynomial contrasts for linear and quadratic trends. Expected treatment means of milk yield were 34.8, 36.8, and 38.8 kg/d, and error variance (σ^2^ = 9.99 kg^2^/d^2^) was derived from the SEM reported by Boerman et al. (2015). In a replicated Latin square with 4 squares (12 cows total, 4 per treatment, 36 experimental units), the power to detect the linear contrast at α = 0.05 was 0.83. Normality of residuals and homogeneity of variance were assessed for each continuous dependent variable after fitting the statistical model, and none of the response variables violated the normality assumption. Data were analyzed by ANOVA with linear mixed model using the MIXED procedure of SAS (version 9.4, SAS Institute Inc.) using the following model:

*Y_ijkl_* = µ + *T_i_* + *P_j_* + *S_k_* + *T_i_* × *P_j_* + *T_i_* × *S_k_* + *P_j_* × *S_k_* + *C*(*S*)*_lk_* + *e_ijkl_*,

where *Y_ijkl_* is the dependent variable, *μ* is the overall mean, *T_i_* is the fixed effect of treatment (*i* = 1 to 3), *P_j_* is the fixed effect of period (*j* = 1 to 3), *S_k_* is the fixed effect of square (*k* = 1 to 4), *T_i_* × *P_j_* is the interaction between *T_i_* and *P_j_*, *T_i_* × *S_k_* is the interaction between *T_i_* and *S_k_*, *P_j_* × *S_k_* is the interaction between *P_j_* and *S_k_*, *C*(*S*)*_lk_* is the random effect of cow (*l* = 1 to 3) nested within square (*k* = 1 to 4), and *e_ijkl_* is the random residual. In all mixed models, the Kenward-Roger method was used to approximate the denominator df for the *F* tests. The covariance structure was modeled based on fit statistics using the smallest corrected Akaike information criterion (**AIC**). The AR(1) covariance structure produced the lowest AIC and was used for all variables. In all analyses, single df orthogonal polynomial contrasts included to determine linear and quadratic effects of treatments on the response analyzed. The interactive matrix language procedure of SAS (PROC IML) was used to generate the orthogonal coefficients to adjust for the unequal spacing of starch in treatments. The resulting coefficients for LS, MS, and HS were, respectively, −0.735, 0.059, and 0.676 for the linear contrast, and 0.356, −0.814, and 0.458 for the quadratic contrast. Data are reported as LSM and SEM. Evidence of statistical significance against the null hypothesis was considered at *P* ≤ 0.05, and tendency was considered at 0.05 < *P* ≤ 0.10.

All 12 cows completed the 63-d experimental period and contributed data for statistical analysis. Data on production performance and feed efficiency are presented in Table 2. Increasing dietary starch concentration linearly (*P* ≤ 0.03) increased net energy intake (38.9 vs. 40.3 vs. 40.7 ± 0.87 Mcal/d) and milk yield (33.5 vs. 36.6 vs. 38.9 ± 1.24 kg/d), and tended (*P* = 0.07) to linearly increase ECM yield (39.1 vs. 43.5 vs. 43.1 ± 1.50 kg/d), without a concurrent change in BW, BW change, or DMI, resulting a tendency for improved feed efficiency (ECM/DMI; 1.62 vs. 1.77 vs. 1.78 ± 0.06 kg/kg; *P* = 0.06) in dairy cows. Milk fat concentration tended to decrease linearly (*P* = 0.08), from 4.45% in LS to 4.27% in HS. True protein concentration responded quadratically (*P* < 0.01), with higher values for MS (3.64%) and HS (3.61%) compared with LS (3.44%). Lactose concentration increased linearly (*P* = 0.02), from 4.92% in LS to 4.98% in HS. Fat and lactose yields were unaffected by dietary starch level, whereas true protein yield increased linearly (*P* = 0.05), with values of 1.17, 1.37, and 1.34 ± 0.06 kg/d for LS, MS, and HS, respectively. Milk urea nitrogen decreased linearly (*P* < 0.01) from 18.1 mg/dL in LS to 10.4 ± 0.56 mg/dL in HS-fed cows. Results on whole-body nutrient metabolism are summarized in Table 3. Respiratory quotient showed a tendency (*P* = 0.06) for a quadratic pattern, as cows fed LS and MS had similar RQ (LS = 0.90 vs. MS = 0.90 ± 0.01), whereas cows fed HS had an RQ of 0.94 ± 0.01, reflecting a metabolic shift toward greater carbohydrate oxidation. Net carbohydrate oxidation rose linearly (*P* < 0.01), increasing from 5,432 to 7,276 ± 366 g/d, and tended (*P* = 0.09) toward a quadratic effect when adjusted for mBW, rising from 39.0 to 52.0 ± 2.6 g/kg mBW for LS and HS, respectively. In contrast, FOX_net_ exhibited a quadratic response (*P* = 0.03), peaking in MS-fed cows and declining in HS-fed cows, with values of 742, 906, and 450 ± 150 g/d for LS, MS, and HS, respectively. A similar pattern was observed when FOX_net_ was normalized to mBW. Heat production increased linearly (*P* < 0.01), rising from 34.8 to 38.1 ± 0.73 Mcal/d, and showed a similar linear increase when expressed per unit of mBW for LS and HS, respectively.

**Table 2:** Effect of dietary starch level on DMI, productive performance, and feed efficiency in mid-lactating Holstein cows

Item	Treatment^1^			SEM	Polynomial contrast^2^	
	LS	MS	HS		Linear	Quadratic
BW, kg	726	733	724	9.5	0.90	0.13
BW change,^3^ kg/d	0.70	−0.82	0.39	0.87	0.70	0.17
DMI, kg/d	24.3	24.9	24.7	0.53	0.40	0.35
Net energy intake, Mcal/d	38.9	40.3	40.7	0.87	0.03	0.52
Concentration, %						
Fat	4.45	4.47	4.27	0.11	0.08	0.17
True protein	3.44	3.64	3.61	0.06	<0.01	<0.01
Lactose	4.92	4.92	4.98	0.05	0.02	0.07
Yield, kg/d						
Milk	33.5	36.6	38.9	1.24	<0.01	0.83
Fat	1.52	1.68	1.60	0.08	0.51	0.30
True protein	1.17	1.37	1.34	0.06	0.05	0.19
Lactose	1.69	1.86	1.86	0.07	0.15	0.44
ECM	39.1	43.5	43.1	1.50	0.07	0.27
MUN, mg/dL	18.1	12.9	10.4	0.56	<0.01	0.07
Feed efficiency, kg/kg						
ECM:DMI	1.62	1.77	1.78	0.06	0.06	0.43

1 Mid-lactating Holstein cows were fed diets containing low starch (LS), medium starch (MS), or high starch (HS).

2 Linear = linear effect of dietary starch; quadratic = quadratic effect of dietary starch.

3 Body weight change was calculated during the last day of the sampling week in each period.

**Table 3:** Effect of dietary starch level on nutrient partitioning in mid-lactating Holstein cows

Item	Treatment^1^			SEM	Polynomial contrast^2^	
	LS	MS	HS		Linear	Quadratic
Respiratory quotient	0.90	0.90	0.94	0.01	0.02	0.06
COX_net_, g/d	5,432	5,848	7,276	366	<0.01	0.11
COX_net_, g/kg mBW	39.0	41.6	52.0	2.6	<0.01	0.09
FOX_net_, g/d	742	906	450	150	0.11	0.03
FOX_net_, g/kg mBW	5.28	6.42	3.29	1.07	0.13	0.03
HP, Mcal/d	34.8	37.4	38.1	0.73	<0.01	0.20
HP, Mcal/kg mBW	0.25	0.26	0.27	0.01	<0.01	0.36

1 Mid-lactating Holstein cows were fed diets containing low starch (LS), medium starch (MS), or high starch (HS).

2 Linear = linear effect of dietary starch; quadratic = quadratic effect of dietary starch.

Increasing dietary starch concentration in mid-lactation dairy cows resulted in coordinated shifts in whole-body energy metabolism and productive performance, demonstrating that graded starch supplementation can effectively modulate nutrient partitioning to support lactation. Despite similar BW and DMI across treatments, starch enrichment increased net energy intake, which was reflected in greater milk and ECM yield. The increase in ECM highlights the ability of HS diets to direct nutrients toward productive functions, indicating improved feed efficiency, and this observation is consistent with the findings of Boerman et al. (2015). Results from this study further suggest that the HS diet may have selected for a ruminal microbial community enriched in propionate-producing bacteria (Li et al., 2022). The greater propionate production would increase its absorption and delivery to the liver, where propionate serves as the primary substrate for hepatic gluconeogenesis. The resulting increase in glucose availability supports greater lactose synthesis, which is the key osmotic driver of milk yield. This enhanced glucose supply also contributes to a broader “carbohydrate sufficiency” signal transmitted through gut-liver, gut-pancreas, and gut-peripheral tissue axes. Consistent with this, propionate-derived glucose in response to increased dietary starch may stimulate insulin secretion (Zhang et al., 2015; Piccioli-Cappelli et al., 2022), and elevated insulin provides a systemic anabolic signal that attenuates lipid mobilization and redirects conserved and newly synthesized energy toward the mammary gland (Peters and Elliot, 1984). In this study, DMI was not affected by the treatments, which is consistent with observations by Allen et al. (2009), who reported that feeding cows higher amounts of starch increased ruminal propionate concentrations, leading to smaller meal sizes and reduced DMI. In contrast, the LS diet contained substantially more forage-derived NDF, which can slow digesta passage and increase rumen fill (Salfer et al., 2018), potentially limiting intake. Notably, increasing dietary starch leads to reduced CH_4_ emissions (Juckem et al., 2025), largely because propionate serves as a hydrogen sink. Thus, it is plausible that improvement in energetic efficiency primarily reflects shifts in rumen fermentation and substrate metabolism, with CH_4_ reduction occurring in parallel rather than directly driving milk synthesis. The effect of starch on protein metabolism was also evident, though secondary to the overarching energetic response. Higher starch intake was associated with increased milk protein concentration and yield, reflecting enhanced ruminal microbial protein synthesis supported by improved synchronization of energy and N availability. The observed decline in MUN with increasing dietary starch further supports reduced ammonia losses and more efficient N capture (Al-Dehneh et al., 1997), indicating coordinated partitioning of both energy and N toward productive functions.

In this study, whole-body energetics measurements provide mechanistic insight into the observed production responses. The linear increase in COX_net_ with increasing starch indicates a direct shift toward glucose-driven energy metabolism. In contrast, FOX_net_ exhibited a quadratic response, peaking at intermediate starch intake and declining at the highest starch level, suggesting progressive displacement of lipid catabolism as carbohydrate availability increased. These patterns are consistent with the proposed endocrine and metabolic signaling framework in which insulin and propionate act as key regulators of substrate selection and nutrient partitioning (Peters and Elliot, 1984; Allen et al., 2009; Zhang et al., 2015). Consistent with these changes, the increased RQ observed with the HS diet likely reflects a preferential oxidation of carbohydrates over lipids to support the energetic demands of lactogenesis. Heat production, both in absolute terms and relative to mBW, increased linearly with dietary starch, paralleling the rise in ECM. Although increasing dietary starch has been shown to increase BW gain in early- and late-lactation cows (Piccioli-Cappelli et al., 2022), we did not observe effects on BW or BW change in this study. However, it is plausible to indicate that the increases in COX_net_, RQ, and HP indicate that the additional starch may have been utilized through enhanced carbohydrate oxidation to support lactational metabolism rather than body tissue retention. Overall, these findings demonstrate that increasing dietary starch enhances efficiency by promoting carbohydrate oxidation and directing energy toward milk synthesis in dairy cows.
